# Estrogen Metabolite 2-Methoxyestradiol Attenuates Blood Pressure in Hypertensive Rats by Downregulating Angiotensin Type 1 Receptor

**DOI:** 10.3389/fphys.2022.876777

**Published:** 2022-05-02

**Authors:** Yong Zhang, Benard O. Ogola, Laxmi Iyer, Vardan T. Karamyan, Thomas Thekkumkara

**Affiliations:** ^1^ Department of Pharmaceutical Sciences, Texas Tech University Health Sciences Center School of Pharmacy, Amarillo, TX, United States; ^2^ Department of Pharmacology, Tulane University, New Orleans, LA, United States

**Keywords:** heart rate, hypertension, 2-methoxtestradiol, renin angiotensin system, angiotensin type 1 receptor

## Abstract

The therapeutic potential of 2-Methoxyestradiol (2ME2) is evident in cardiovascular disease. Our laboratory has previously demonstrated the mechanism involved in the 2ME2 regulation of angiotensin type 1 receptor (AT1R) *in vitro*. However, 2ME2 regulation of angiotensin receptors and its effects on blood pressure (BP) and resting heart rate (RHR) are uncertain. In this study, male and female Wistar-Kyoto (WKY) rats infused with angiotensin II (65 ng/min) and male spontaneously hypertensive rats (SHR) were surgically implanted with telemetric probes to continuously assess arterial BP and RHR. In both male and female WKY rats, 2ME2 treatment (20 mg/kg/day for 2 weeks) resulted in a significant reduction of Ang II-induced systolic, diastolic, and mean arterial BP. Moreover, significant weight loss and RHR were indicated in all groups. In a separate set of experiments, prolonged 2ME2 exposure in male SHR (20 mg/kg/day for 5 weeks) displayed a significant reduction in diastolic and mean arterial BP along with RHR. We also found downregulation of angiotensin receptors and angiotensinogen (AGT) in the kidney and liver and a reduction of plasma Ang II levels. Collectively, we demonstrate that 2ME2 attenuated BP and RHR in hypertensive rats involves downregulation of angiotensin receptors and body weight loss.

## Introduction

The renin-angiotensin system (RAS) plays an important role in maintaining blood pressure by regulating water and sodium homeostasis (Sparks et al., 2014). Angiotensin II (Ang ll) stimulates angiotensin type 1 receptor (AT1R) and is responsible for the downstream functions in the cardiovascular system (Crowley et al., 2006). Intra-tissue Ang ll/AT1R activation is involved in tissue fibrosis and remodeling in the cardiovascular tissues (Crowley et al., 2005). Dysregulation of Ang ll/AT1R signaling cascade promotes essential and secondary hypertension as well as hypertension-related cardiovascular disease (CVD) (Yamamoto et al., 2006). Pharmacological interventions targeting Ang ll production or AT1R activation are widely-used for the treatment of hypertension and related CVD (Paz Ocaranza et al., 2020). However, challenges still remain in the treatment and management of hypertension and associated CVD.

Female sex hormones particularly estradiol protects against hypertension and related CVD, however, a randomized clinical trial of hormone replacement therapy with estrogen and/or progesterone did not indicate significant benefits in postmenopausal women (Manson et al., 2003). Estrogen metabolism involves CYP450 enzymes in hepatic and extrahepatic tissues yielding 2-hydroxyestradiol and 2-methoxyestradiol (2ME2) generated by catechol-O-methyltransferase (COMT) enzymes (Tsuchiya et al., 2005). 2ME2 binds to G protein-coupled estrogen receptor, although with a lower affinity compared with estradiol, and activates downstream signaling pathways (Koganti et al., 2012; Koganti et al., 2014; Samartzis et al., 2016; Schaufelberger et al., 2016; Ogola et al., 2018; Singh et al., 2020). Clinical observations indicate an inverse relationship of plasma 2ME2 level and preeclampsia (Barnea et al., 1988; Kanasaki et al., 2008; Zhang et al., 2014; Wantania et al., 2017). Additionally, preclinical studies demonstrate that *in vitro* exposure to 2ME2 suppresses human vascular smooth muscle cell (SMC) proliferation, downregulation of AT1R, and relaxation of pre-contracted aortic rings (Barchiesi et al., 2002; Gui et al., 2008; Koganti et al., 2012; Koganti et al., 2014). *In vivo* administration of 2ME2 blunts Ang II-induced hypertension in Cyp1b1-null mice and deoxycorticosterone acetate salt-induced hypertension in Wistar rats (Tofovic et al., 2006). 2ME2 vasoprotective effect involves increasing nitric oxide synthase and inhibiting coronary artery remodeling (Tofovic et al., 2005; Bonacasa et al., 2008; Pingili et al., 2017). Here, we report the impact of 2ME2 on arterial BP and resting heart rate (RHR) in Ang II-induced hypertension and spontaneously hypertensive rats. Our study shows that 2ME2 reduces BP, RHR, and body weight, and downregulates angiotensin receptors.

## Materials and Methods

### Telemetry Probe Implantation, Treatments, and Blood Pressure Measurements

Animal studies were approved by the Institutional Animal Care and Use Committee of Texas Tech University Health Sciences Center. A total of 10 male and 10 female Wistar Kyoto (WKY) rats as well as 10 spontaneously hypertensive rats male (SHRs) at 15 weeks of age were obtained from the Charles River (San Diego, CA). After 1 week of acclimation, animals were surgically implanted with PA-C40 telemetries (Data Sciences International, St. Paul, MN) and allowed to recover for 10 days before continuous monitoring of systemic BP and heart rate with recording at 5-min intervals recording for 24 h. Day average blood pressure was used as final readings. Male and female WKY rats were randomly divided into two groups of 5, respectively. Each group received either pretreatment with vehicle (polyethylene glycol 400) (Al Shoyaib et al., 2019) or 2ME2 (20 mg/kg, daily intraperitoneal injection) for 1 week a dose shown to have significant effects on the body and uterine weights (Sibonga et al., 2003). At 1 week after administration of 2ME2, ALZET osmotic pumps (model 2002, Cupertino, CA) were implanted subcutaneously to start to infuse Ang II (Bachem Americas, Torrance, CA) at a rate of 65 ng/min. The Ang II-infused animals continued to receive vehicle or 2ME2 treatments for another week before euthanasia. For the study with SHRs, the animals were randomly divided into two groups of 5 after telemetry probe implantation and received vehicle or 2ME2 treatment (20 mg/kg, daily intraperitoneal injection) for five consecutive weeks. Body weights were recorded every 3 days for WKY rats and weekly for SHR. After euthanasia, end-organ tissues were collected for molecular and biochemistry assays.

### Radioactive Receptor Binding Assay

Crude plasma membrane preparation was prepared as previously described (Aiyar et al., 1993). Briefly, tissues were homogenized with a polytron in the binding buffer (50 mm Tris-HC1 pH 7.4, 120 mm NaCl, 4 mm KCl, 1 mm CaC12, 10 μg/ml bacitracin, 0.25% BSA, 2 mg/ml dextrose and proteinases inhibitors). The homogenate was centrifuged at 1000 g for 7 min, and then the supernatant was centrifuged at 45,000 g for 20 min. The membrane pellets were resuspended in the binding buffer. Protein concentrations were determined using the Bio-Rad protein assay system based on the Bradford method.

Equally aliquoted in triplicate, 250 µg of membrane preparation in binding buffer was incubated with 0.1 nM [^3^H]Ang ll for total binding and the other portion for specific binding with 1 µM of unlabeled Ang ll for 15 min at room temperature followed by an addition of 0.1 nM [^3^H]Ang ll in the binding buffer and incubated for additional 30 min. The reaction mixture was filtered through a 0.45 µm nitrocellulose membrane (EMD Millipore, Billerica, MA) to trap the [^3^H]Ang ll-bound membrane preparation in the filter membrane. The nonspecifically bound Ang ll was removed by washing three times with chilled PBS containing calcium and magnesium. The [^3^H]Ang ll-bound filter membrane was transferred to counting vials containing a Scintillation cocktail (Fisher Sci, Waltham, MA). Radioactive disintegration per minute was determined using a Beckman auto-scintillation counter (Beckman Coulter, Brea CA). Specific [^3^H] Ang ll binding was defined as the portion of the total binding displaced by 1 μM unlabeled Ang ll.

The effect of 2ME2 on the AT1R-binding affinity of Ang ll was examined as previously described (Swillens, 1992). Membrane preparation was incubated with unlabeled Ang ll at increased concentrations from 1 p.m. to 10 μM for 20 min before adding 0.1 nM of [^3^H]Ang ll for additional 25 min, and then filtered through 0.45 µm nitrocellulose membrane. After 3 times of wash with PBS [^3^H] Ang ll-bound filter membrane was transferred to counting vials and the radioactive disintegration per minute was determined using a Beckman^®^ scintillation counter. The data were normalized to protein concentrations. Assays were done in triplicate.

### Angiotensin II EIA Assay

Plasma angiotensin II concentrations were measured using Angiotensin II EIA Kit (RAB0010, Sigma-Aldrich, St. Louis, MO) according to the manufactory manual. Briefly, 100 µL of anti-Ang II antibody were added to each well and incubated overnight at 4°C. After washing with washing buffer, 100 µL of plasma or standard samples containing 20 pg/ml of biotinylated Ang II were added to the wells and incubated for 2.5 h at room temperature with gentle shaking. After washing, 100 µL of HRP-streptavidin solution was added to each well and incubated for 45 min, and then 100 µL of TMB one-step substrate reagent was added. After 30 min of incubation, 50 µL of stop solution was added to each well and optical absorbance was read at 450 nm (BioTek SynergyMx). The assays were done in duplicate.

### Renin Activity Assay

Plasma renin activity was measured using a fluorometric Renin Assay Kit (MAK157, Sigma-Aldrich, St. Louis, MO) according to the manufactory manual. Briefly, 50 µL of plasma or standard samples were mixed with equal volumes of assay buffer containing renin substrate. The mixture was incubated for 30–60 min with measurements carried out every 5 min *λ*_ex_ = 540 nm/*λ*_em_ = 590 nm). The assays were done in duplicate.

### Immunoblotting

Tissues were lysed in 50 mM HEPES, 1% Triton X-100, 50 mM NaCl, 50 mM NaF, 10 mM sodium pyrophosphate, 5 mM EDTA buffer containing protease and phosphatase inhibitors. Protein lysate estimation was done using the Bradford method followed by 50 µg that was aliquoted and added to a loading dye and reducing agent. The proteins were then resolved in 8% SDS-PAGE and transferred onto a nitrocellulose membrane (catalog no. 1620115 Bio-Rad Laboratories). The membrane was blocked in 5% non-fat milk in Tris-buffered saline containing 20 mM Tris, pH 7.5, 150 mM NaCl, 0.1% (wt/vol) Tween 20 for 2 h. The membrane was then incubated overnight with monoclonal anti-angiotensinogen (AGT) antibody (1:1,000 dilution; cat#77 Swant^®^, Switzerland) (Clauser et al., 1984) followed by a wash in TBST and incubated in anti-mouse HRP linked antibody (1:2000 dilution; cat#7076S, Cell Signaling Technology) for 1 h. For loading control, we used GAPDH (primary antibody at 1:1,000 dilution, cat#2118S; secondary antibody at 1:2000 dilution, cat#7074S from Cell Signaling Technology). The membranes were then washed for 10 min three times with TBST at room temperature and exposed to Chemiluminescent Substrate from Thermo Scientific (Thermo-Scientific Pierce, Rockford, IL). Immunoreactive bands were visualized by autoradiography and quantitated using ImageJ.

### Statistical Analysis ANOVA Needed

Statistical analysis was performed using Prism 8.4 (GraphPad Software). Outliers were removed by the ROUT method, whereby Q = 1. The 5 min for 24 h daily blood pressure values were averaged for daytime only, and data were subjected to 2-way ANOVA repeated measures followed by Sidak’s multiple comparisons test. Radio-ligand binding, immunoblot, and heart weight data were analyzed by student *t*-test. The competitive binding assay was analyzed by nonlinear regression. Data are presented as the mean ± SEM and values with *p* < 0.05 were considered statistically significant.

## Results

### 2ME2 Reduces Ang II-Induced Blood Pressure and Heart Rate in Male WKY Rats

**FIGURE 1:**
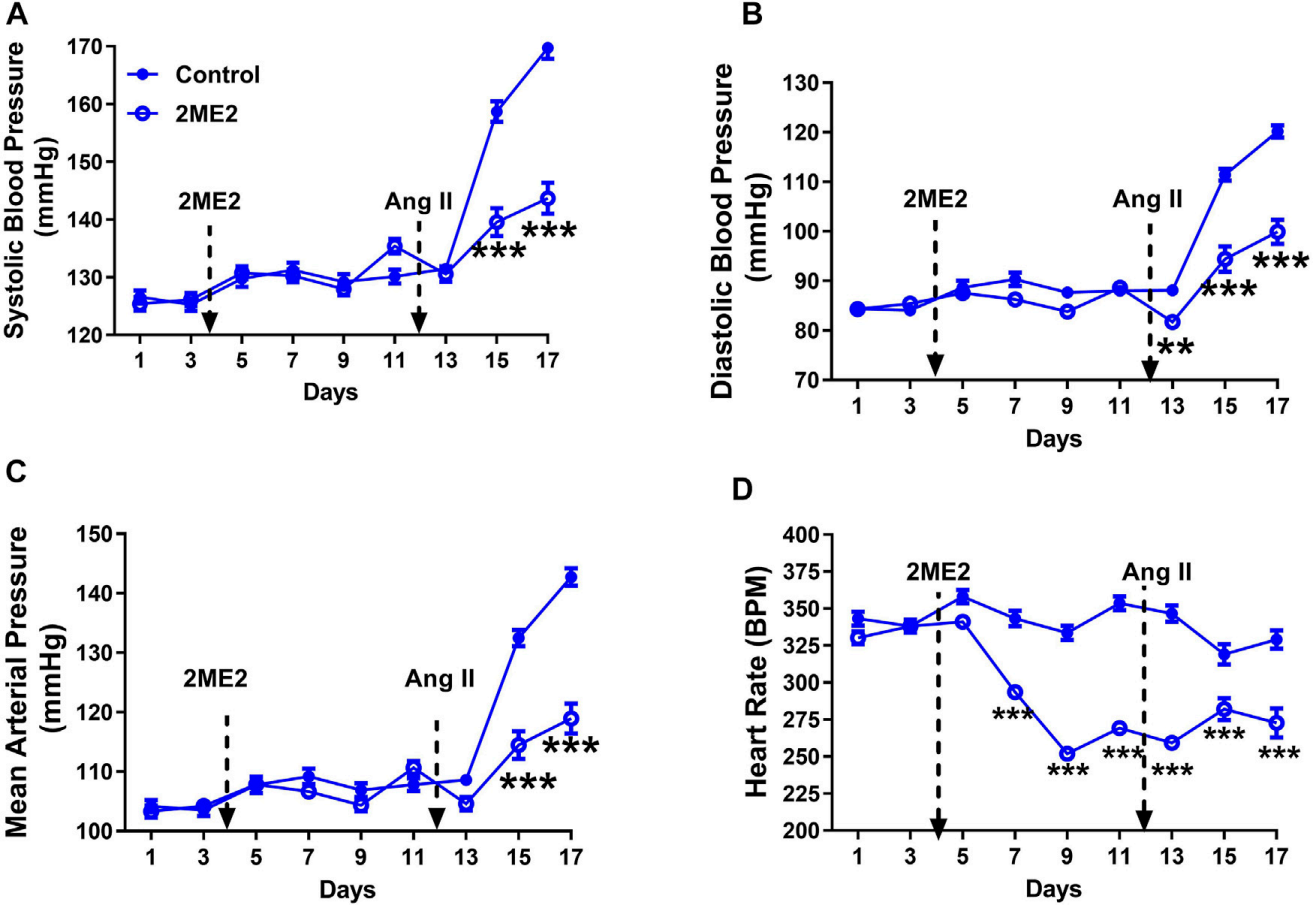
2ME2 reduces Ang II-induced blood pressure and heart rate in male WKY rats. Ang II-induced hypertension is significantly attenuated by 2ME2 treatment in **(A)** systolic **(B)** diastolic, and **(C)** mean arterial pressure **(D)** 2ME2 decreases heart rate prior to Ang II-induced hypertension. The data is shown as Mean ± SEM (2-way ANOVA repeated measures followed by Sidak’s multiple comparison test, **p < 0.01 and ***p < 0.001; *n* = 5).

To determine the impact of 2ME2 treatment in BP, we used Ang II-induced hypertension model in male WKY rats. The rats were administered with either 2ME2 or vehicle for 1 week, followed by five additional days during Ang II administration. In male WKY rats, daytime baseline BP, and RHR were not different between the treatment and control groups before 2ME2 administration (Figure: A. systolic BP; 125 ± 1 vs. 126 ± 1 mmHg, B. diastolic BP; 84 ± 1 vs. 85 ± 1 mmHg, C. mean BP; 104 ± 1 vs. 104 ± 1 mmHg). Similarly, 2ME2 treatment did not induce significant changes in blood pressure by day 11 prior to Ang II infusion (Figure 1: A. systolic BP; 130 ± 1 vs. 135 ± 1 mmHg, B. diastolic BP; 88 ± 1 vs. 89 ± 1 mmHg, C. mean BP; 108 ± 1 vs. 111 ± 1 mmHg *p* > 0.05). Compared with control group, 2ME2 treatment decreased heart rate significantly (354 ± 5 vs. 269 ± 3 BPM; *p* < 0.001, Figure 1D). Notably, Ang II-induced hypertension was substantially blunted by 2ME2 treatment by day 15 and was maintained until day 17 (Figure 1: A. systolic BP; 159 ± 2 vs. 140 ± 2 mmHg, B. diastolic BP; 111 ± 1 vs. 94 ± 3 mmHg, and C. mean BP; 132 ± 1 vs. 114 ± 2; *p* < 0.001). Concurrently, rats treated with 2ME2 continuously indicated decreased heart rate (319 ± 7 vs. 282 ± 7 BPM; *p* < 0.001, Figure 1D).

### 2ME2 Decreases Ang II-Induced Blood Pressure and Heart Rate in Female WKY Rats

**FIGURE 2:**
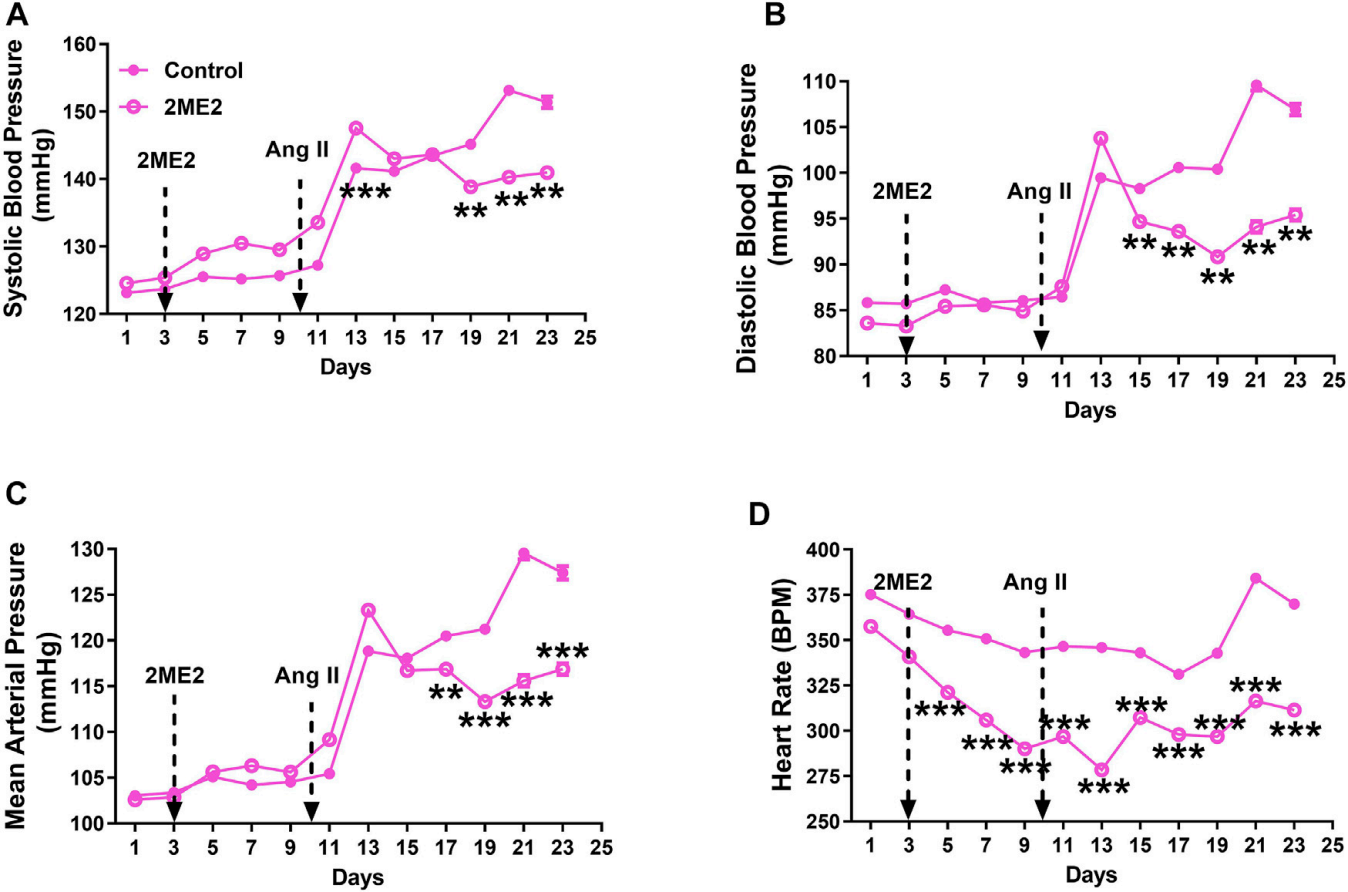
2ME2 decreases Ang II-induced blood pressure and heart rate in female WKY rats. Ang II-induced hypertension is significantly attenuated by 2ME2 treatment in **(A)** systolic **(B)** diastolic **(C)** mean and arterial pressure **(D)** 2ME2 decreases heart rate prior to Ang II-induced hypertension. The data is shown as Mean ± SEM (2-way ANOVA repeated measures followed by Sidak’s multiple comparison test, **p < 0.01 and ***p < 0.001; *n* = 5).

To determine whether 2ME2 impact on blood pressure is dependent on sex, we used Ang II-induced hypertension in female rats. While male rats had a significant 2ME2 mediated decrease in SBP after 5 days of Ang II-induced hypertension, female rats required a longer time (13 days) for a similar observational effect on BP. Baseline BP was not different between control and treatment group (Figure 2: A. systolic BP; 123 ± 1 vs. 125 ± 1 mmHg, B. diastolic BP; 86 ± 1 vs. 84 ± 1 mmHg, C. mean BP; 102 ± 1 vs. 103 ± 1 mmHg *p* > 0.05). However, 2ME2 induced blood pressure increase on day 13 (Figure 2: A. systolic BP; 142 ± 1 vs. 147 ± 1 mmHg, B. diastolic BP; 99 ± 1 vs. 104 ± 1 mmHg, C. mean BP; 119 ± 1 vs. 123 ± 1 mmHg; *p* < 0.01). On day 23, 2ME2 had significantly lowered Ang II-induced hypertension (Figure 2: A. systolic BP; 151 ± 1 vs. 141 ± 1 mmHg, B. diastolic BP; 107 ± 1 vs. 95 ± 1 mmHg, C. mean BP; 127 ± 1 vs. 117 ± 1 mmHg; *p* < 0.01). Although 2ME2 attenuated Ang II-induced hypertension, heart rate in 2ME2 treated group versus control consistently decreased from day 1 (375 ± 1 vs. 358 ± 1 BPM; *p* < 0.001) up to day 23 (370 ± 1 vs. 311 ± 1 BPM; *p* < 0.001) independent of Ang II infusion.

### 2ME2 Impacts Blood Pressure and Heart Rate in SHR Rats

**FIGURE 3:**
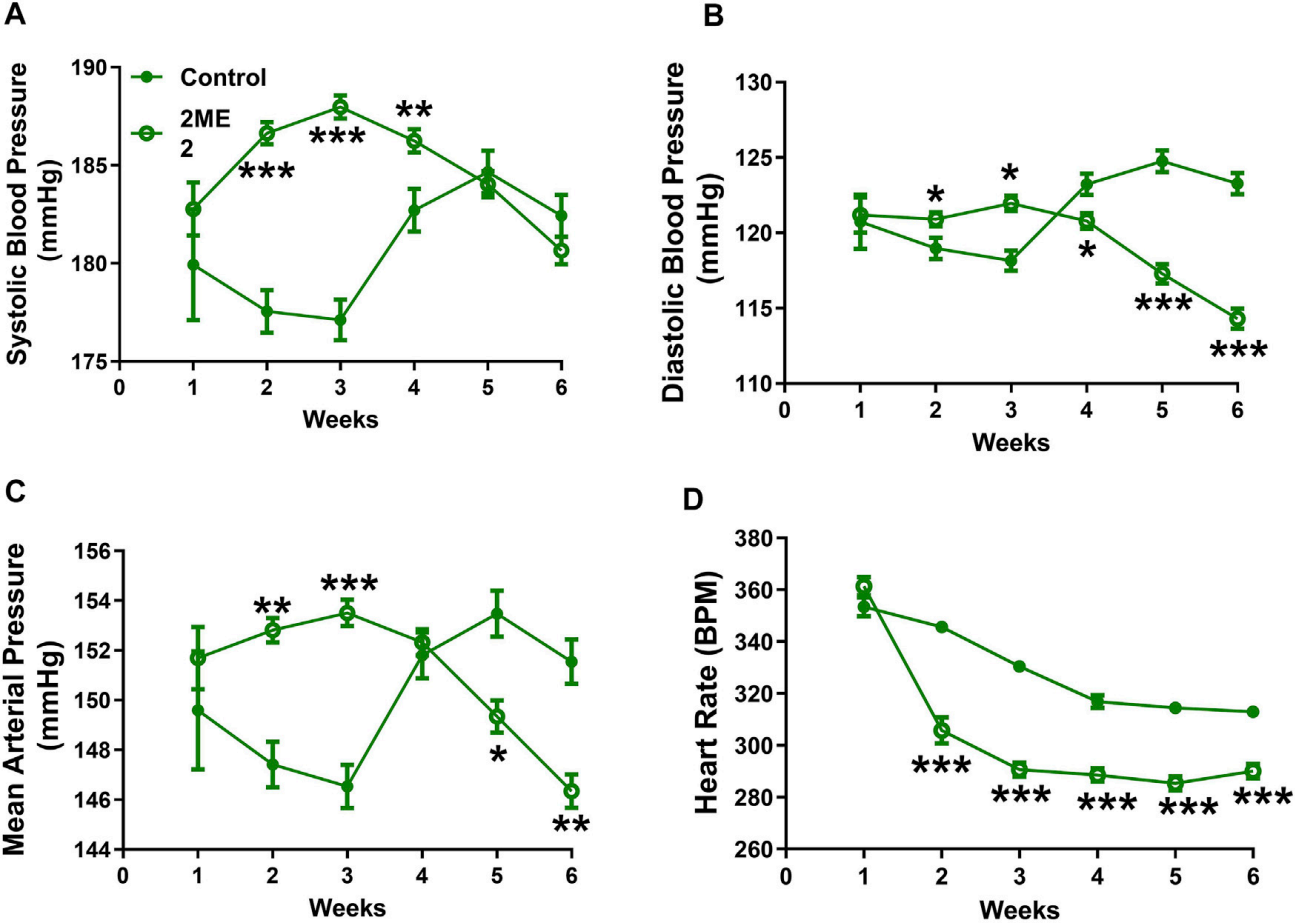
2ME2 impacts blood pressure and heart rate in SHR rats. 2ME2 treatment increased **(A)** systolic BP at the beginning but was unchanged at the end of week 6. **(B)** At the end of the study 2ME2 decreased diastolic **(C)** mean arterial blood pressures and **(D)** heart rate consecutively during treatment. The data is shown as Mean ± SEM (2-way ANOVA repeated measures followed by Sidak’s multiple comparison test, *p < 0.05, **p < 0.01, and ***p < 0.001; *n* = 5).

To determine whether 2ME2 regulates blood pressure in an Ang II hypertension independent model, we used spontaneously hypertensive rats (SHR). Baseline BP was similar at week 1 between the control and 2ME2 treatment group. However, 2ME2 increased SBP in weeks 2 (178 ± 1 vs. 187 ± 0.6 mmHg; *p* < 0.001) and 3 (177 ± 1 vs. 188 ± 0.6 mmHg; *p* < 0.001) before lower recordings were indicated after week 5 (Figure 3: A. systolic BP; 185 ± 0.5 vs. 184 ± 1 mmHg; *p* = 0.6, B. diastolic BP; 125 ± 1 vs. 117 ± 1 mmHg; *p* < 0.001, and C. mean BP; 153 ± 1 vs. 149 ± 1 mmHg: *p* = 0.006). Similar to WKY rats, 2ME2 mediated decrease in the SHR heart rate after week 1 (346 ± 2 vs. 306 ± 5 BPM; *p* < 0.0001, Figure 3D) that was sustained until week 6 (313 ± 2 vs. 290 ± 3 BPM; *p* < 0.0001, Figure 3D).

### 2ME2 Downregulates AT1R Expression in Liver and Kidney of WKY and SHR

**FIGURE 4:**
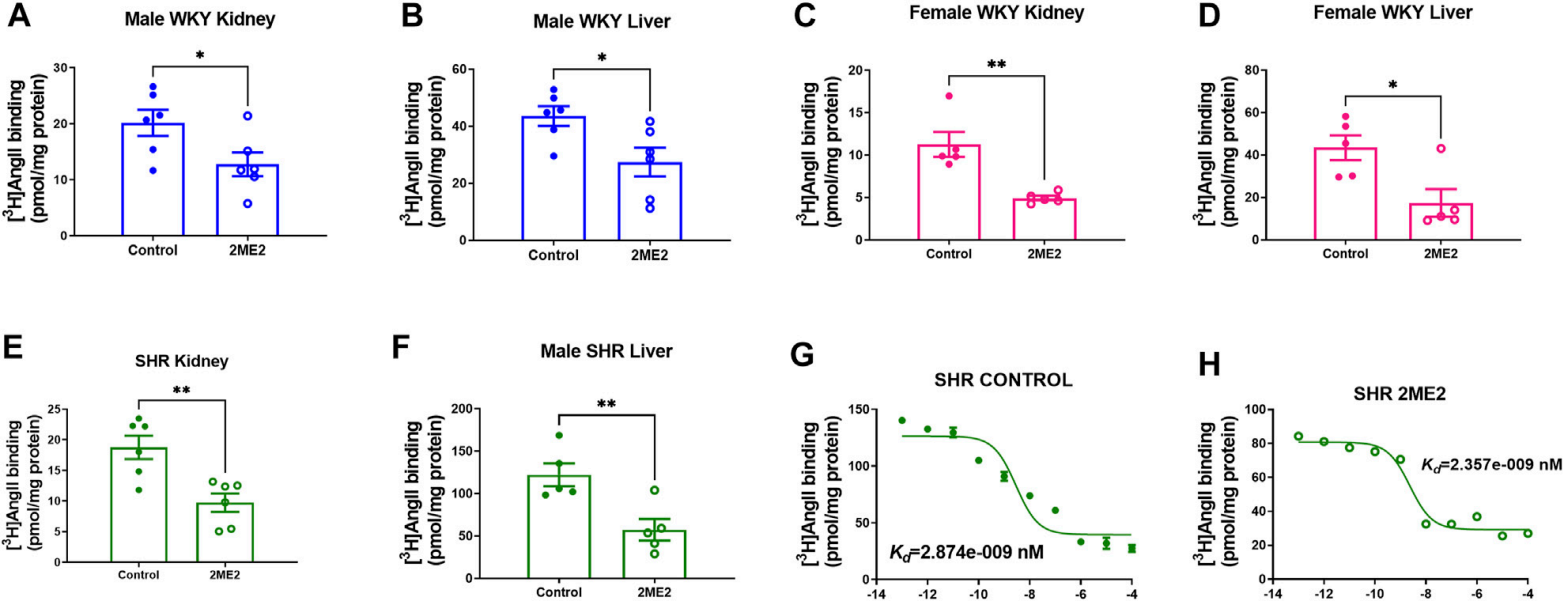
2ME2 attenuates AT1R expression in kidney and liver of WKY and SHR. Unpaired *t* test indicates decrease AT1R expression after 2ME2 treatment in male WKY **(A)** kidney (*p* = 0.04) and **(B)** liver (*p* = 0.02) and female WKY **(C)** kidney (*p* = 0.003) and **(D)** liver (*p* = 0.02). **(E)** 2ME2 similarly decreased AT1R in kidney (*p* = 0.004) and **(F)** liver (*p* = 0.008) of SHR. Non-linear fit regression analysis shows Ang II affinity to AT1R in SHR liver is not impacted by **(G,H)** 2ME2 treatment (*p* = 0.7).

A radio-ligand binding assay was used to determine angiotensin receptors expression in liver and kidney cortex membrane preparation. The data indicated a significant decrease of Ang II binding in the 2ME2-treated group compared to vehicle in male WKY kidney (20 ± 2 vs. 13 ± 2 pmol/mg protein; *p* = 0.04, Figure 4A) and liver (44 ± 3 vs. 27 ± 5 pmol/mg protein; *p* = 0.02, Figure 4B). Similar findings were observed in female WKY rat’s kidney (11 ± 1 vs. 5 ± 0.2 pmol/mg protein; *p* = 0.003, Figure 4C) and liver (43 ± 6 vs. 17 ± 6 pmol/mg protein; *p* = 0.02, Figure 4D). In SHR, AT1R expression was significantly decreased by 2ME2 treatment in kidney (19 ± 2 vs. 10 ± 2 pmol/mg protein; *p* = 0.004, Figure 4E) and liver (122 ± 13 vs. 57 ± 13 pmol/mg protein; *p* = 0.008, Figure 4F) tissues. Compared to control (K_*d*_ = 2.87 nM; Figure 4G), 2ME2 (K_*d*_ = 2.36; Figure 4H) treatment did not alter Ang II binding affinity to AT1R (*p* = 0.65).

### 2ME2 Impacts Renin-Angiotensin System Proteins in SHR

**FIGURE 5:**
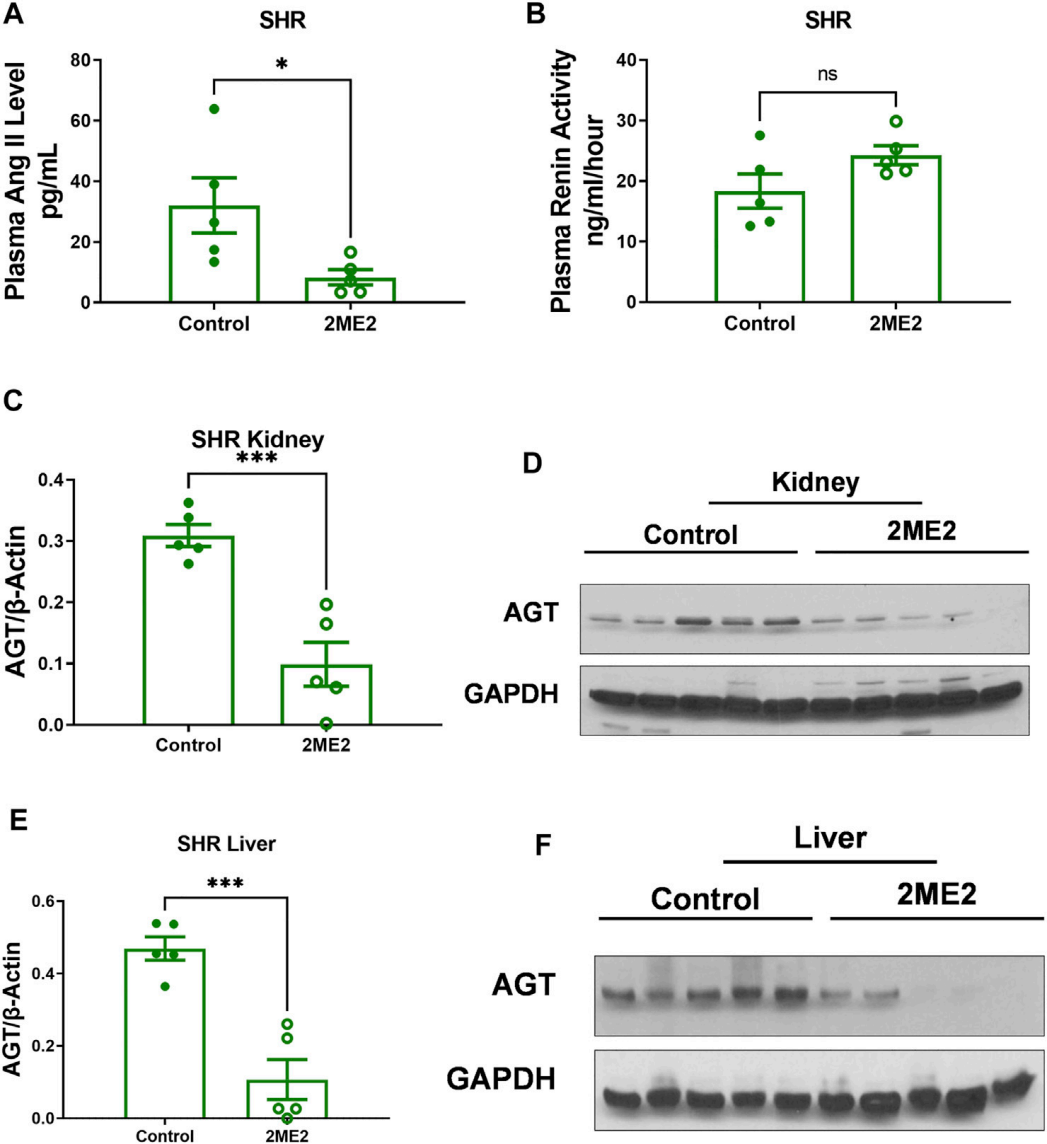
2ME2 impacts RAS proteins in SHR. T-tests indicated that **(A)** 2ME2 reduced plasma Ang II (*p* = 0.04) but not **(B)** renin activity level. Immunoblot analysis with representative blot indicates 2ME2 decreases angiotensinogen (AGT) protein (*p* < 0.001) in kidney **(C,D)** and liver (*p* < 0.01) last sample was eliminated as outlier **(E,F)**.

To determine whether the prolonged effect of a 2ME2-mediated decrease in blood pressure in SHR is associated with changes in renin-angiotensin proteins, we assessed circulating plasma Ang II that was decreased (32.1 ± 9.1 vs. 8.3 ± 2.5 pg/ml, n = 5; *p* = 0.04, Figure 5A) whereas renin activity trended upwards though lacked statistical significance (18.4 ± 2.8 vs. 24.3 ± 1.6 ng/ml/hour; *p* = 0.1, Figure 5B). Immunoblot analysis of angiotensinogen (AGT) in 2ME2 treated SHR was significantly decreased in the kidney (0.3 ± 0.02 vs. 0.1 ± 0.04; *p* < 0.001, Figures 5C,D) and liver (0.5 ± 0.03 vs. 0.1 ± 0.06; *p* < 0.01, Figures 5E,F) tissues.

### 2ME2 Treatment Results in Body and Heart Weight Loss in WKY and SHR

**FIGURE 6:**
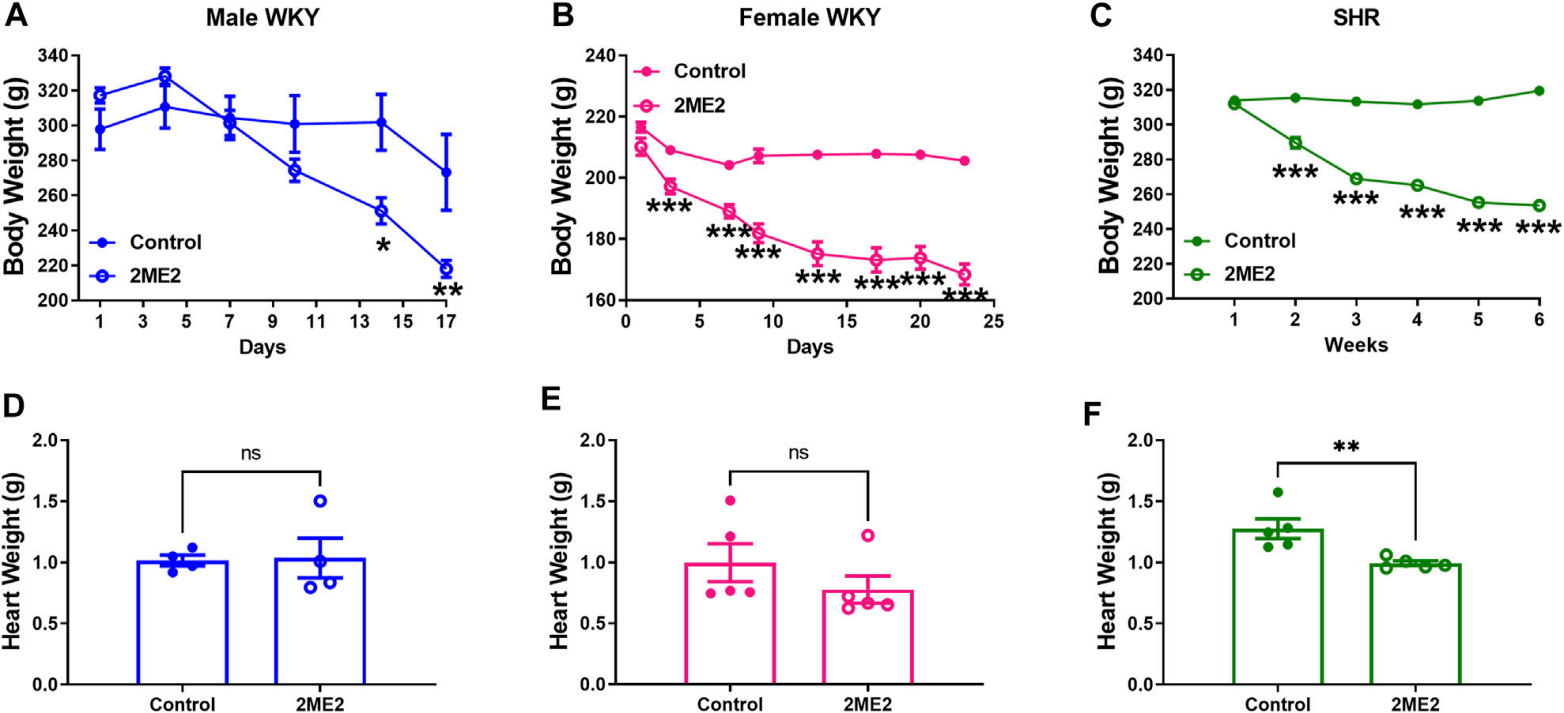
2ME2 treatment results in body and heart weight loss in WKY and SHR. 2 Way ANOVA indicated that **(A)** 2ME2 indicated significant decrease in body weight in male **(B)** and female WKY rats, and **(C)** SHR. Using t-tests, heart weight was not significantly impacted by 2ME2 treatment in **(D)** male **(E)** and female WKY (*p* = 0.9), but **(F)** SHR. (*p < 0.05, **p < 0.01, ***p < 0.001) compared with control.

2ME2 treatment substantially decreased body weight in male (Day 17: 273 ± 22 vs. 218 ± 5 g; *p* = 0.04, Figure 6A) and female WKY male (Day 17: 207 ± 2 vs. 168 ± 3 g; *p* < 0.001, Figure 6B) rats, and SHRs (Week 5: 319 ± 2 vs. 253 ± 1 g; *p* < 0.001, Figure 6C). Heart weight was not significantly impacted by 2ME2 treatment in either male (1.02 ± 0.04 g vs. 1.04 ± 0.2 g; *p* = 0.9, Figure 6D) or female WKY rats (1.0 ± 0.15 g vs. 0.8 ± 0.11 g; *p* = 0.2, Figure 6E), but male SHR indicated significant changes (1.28 ± 0.08 g vs. 1.0 ± 0.02 g; *p* = 0.009, Figure 6F).

## Discussion

In this study, we demonstrated that 2ME2 attenuates Ang II-induced hypertension in WKY rats independent of sex. However, the SHR model of hypertension indicated moderate blood pressure changes to 2ME2 treatment. We have shown that 2ME2 downregulates Ang II receptors expression in the kidney cortex and liver without impacting Ang II binding affinity. By decreasing mean arterial pressure in SHR, 2ME2 also reduced circulating plasma Ang II without impacting renin activity, however, a significant reduction in kidney and liver angiotensinogen was indicated. Interestingly, decreased heart rate in 2ME2 treated rats was independent of Ang II-induced hypertension in WKY rats or the SHR model. Additionally, 2ME2 induced significant weight loss in both male and female WKY and SHR and decreased heart weight in SHR only. The findings hereby highlight and extend previous studies demonstrating the important role of the estrogen metabolite 2ME2 in blood pressure regulation.

Our results are similar to the findings indicating that Ang II-induced hypertension in male and female mice is attenuated by 2ME2 treatment (Pingili et al., 2017). Ang II infusion in null COMT female mice indicates higher blood pressure suggesting that loss of estradiol metabolism pathway is detrimental during hypertension (Jennings et al., 2014). Additionally, the aorta of COMT-deficient mice expresses higher levels of AT1R underscores the significance of catechol estrogens in regulating RAS (Ueki et al., 2017). Reduction of COMT expression in the liver of the SHR model compared with the WKY rat implies that a 2ME2 mediated decrease in blood pressure confers a protective effect due to lower COMT expression (Tsunoda et al., 2003). The underlying mechanisms of 2ME2 mediated decrease in blood pressure are still debatable, however, our results together with previous studies indicate potential beneficial effects of 2ME2.

In COMT-null mice, 2ME2 downregulated Ang II receptors expression in the aorta and reduced Ang II-induced hypertension corroborates our findings in the kidney and liver (Ueki et al., 2017). However, our study shows increased AngII binding in the liver compared to kidney is supported by a previous study showing that in the liver almost all binding is due to AT1R while in the kidney both AT1R and AT2R are involved (Edwards and Aiyar, 1993). Additionally, enhanced metabolism of Ang II ligand may play a role in the differential tissue binding. It is established that the kidney expresses more angiotensin receptors than the liver (Sechi et al., 1996). However, our study indicates that the SHR liver has more tritiated Ang II binding similar to fetal tissue in Sprague Dawley rats suggesting that the SHR liver conforms to fetal like expressing more AT1R than the kidney (Li Yu et al., 2010). Metabolism of estradiol by CYP450 to 2-hydroxyestradiol (2-OHE) does not impact blood pressure, however, conversion of 2-OHE to 2ME2 by COMT mediates a decrease in BP (Ueki et al., 2017). In the present study, 2ME2 treatment decreased systolic, diastolic, and mean arterial BP in Ang II-induced hypertension in WKY rats; however, in SHR, only diastolic BP and mean arterial pressures were impacted after 3 weeks. SHR treated with 2ME2 showed increased systolic BP in weeks 1, 2, and 3 before dipping, which may reflect a feedback mechanism in response to the reduced heart rate and down-regulated RAS reflecting the feedback mechanism in renin producing cells (Neubauer et al., 2018). It is of note that there was a slight but not significant increase in plasma renin activity.

Ang II binding to AT1R is key to downstream signaling that activates intracellular second messengers and gene regulation (Hunyady and Catt, 2006). Given the lack of specificity of AT1R antibodies, we used radiolabeled Ang II which is more robust and sensitive to the receptor expression (Herrera et al., 2013; Nistala et al., 2013; Ogola et al., 2018). Ang II can bind to both AT1R and AT2R, and our previous studies consistently demonstrated that 2ME2 specifically downregulates AT1R and not AT2R without impacting the affinity of Ang II to the receptors (Koganti et al., 2014; Ogola et al., 2018). We have also previously shown that 2ME2 downregulates AT1R *in vitro* which is similar to our *in vivo* findings (Koganti et al., 2012; Koganti et al., 2014; Ogola et al., 2018). SHR is an inbred genetic model of essential hypertension, in which multiple genes are involved in developing increased blood pressure (Lerman et al., 2019). The current study focused on the 2ME2 effect on RAS. It still remains to be determined why Ang II receptor downregulation and blood pressure reduction were prolonged in the SHR versus WKY model.

Resting heart rate plays an important role in CVD, however, results from clinical studies are mixed indicating different ranges of healthy heart rates (Palatini et al., 2006). The HR reduction might be a direct effect of 2ME2 on the heart by inducing parasympathetic activity indicated in gonadectomized mice with decreased norepinephrine after 2ME2 treatment (Singh et al., 2020). Additionally, 2ME2 effects are indicated to be cytostatic than cytotoxic due to the antiapoptotic and antihyperplastic effect on cultured HepG2 cells (El Naga et al., 2009). 2ME2 protective effects are also indicated to prevent left ventricular hypertrophy and pressure overload in rats (Maayah et al., 2018). Increased RHR is associated with ischemic heart disease in the prospective Nord-Trøndelag County Health Study (Nauman et al., 2011). Temporal changes in HR are also associated with mortality, heart attack, and stroke in the Atherosclerosis Risk in Communities Study (Vazir et al., 2018). Additionally, the Systolic Blood Pressure in Intervention Trial indicated higher RHR increased cardiovascular risk (Sobieraj et al., 2021). Therefore, overall suggestion of managing increasing RHR and BP is complex due to lack of clinical guidelines, although the LIFE study shows that increased RHR by 10 beats per minute increases cardiovascular mortality (Okin et al., 2010). The LIFE study also indicated that Atenolol decreased RHR and the Anglo-Scandinavian Cardiac Outcomes Trial showed that atenolol treated patients had fewer major CVD events than subjects treated with amlodipine (Dahlöf et al., 2005). Preclinical experimental approaches to decrease RHR including renal denervation and carotid baroreceptor stimulation can be a beneficial to improving CVD outcomes (Heusser et al., 2010; Böhm et al., 2020). Our study showed that 2ME2 decreased heart rate in both SHR and WKY rat suggests that endogenous estrogen metabolite can be beneficial to decreasing CVD risk.

Although potential beneficial effect of 2ME2 in CVD is evident, its role in postmenopausal women is unexplored (Dubey and Jackson, 2009). The Women’s Health Initiative (WHI) randomized clinical trial on the beneficial effect of estradiol plus progestin in postmenopausal women indicated that overall CVD risk outweighed the benefits (Shufelt et al., 2014). Experts have debated whether the WHI study timing and the route of delivery of the hormone replacement therapy mitigated the beneficial effects (Hodis et al., 2012; Shufelt et al., 2014). Nonetheless, the Nurses’ Health Study indicated the beneficial effect of long-term users of estrogen alone (Grodstein et al., 2001). The timing hypothesis was evaluated in the Kronos Early Estrogen Prevention Study by beginning estradiol replacement in the perimenopause period through an oral or transdermal application that indicated safety without venous thrombosis and improved effect of the hormones in preventing hot flushes and decreasing the development of carotid intima thickness (Miller et al., 2019). Although the beneficial effect of estrogen was investigated in women, age-matched men also indicated an increase in CVD that needed consideration (Benjamin et al., 2019). Therefore, an alternative to estrogen indicating catechol estrogens particularly 2ME2 can benefit males and females without feminizing effects (Josefsson and Tarkowski, 1997; Maayah et al., 2018).

Angiotensinogen (AGT) is the precursor of all angiotensin peptides (Lu et al., 2016a). Suppressing AGT using antisense oligonucleotides has been shown to reduce blood pressure, atherosclerosis, and body weight (Olearczyk et al., 2014; Yiannikouris et al., 2015; Lu et al., 2016b). Consistently, our data demonstrated 2ME2 had a discernable effect on body weight accompanied by the decreased AGT in both SHR and WKY rats. The reduction in body weight is also observed in AGT-knockout mice that have improved glucose utilization and low macrophage accumulation (Yorifuji et al., 2011; LeMieux et al., 2016). Furthermore, dose-response of 2ME2 indicates decreased body weight in ovariectomized rats is aligned with antihypertrophic effects in the SHR (Sibonga et al., 2003).

In summary, for the first time, we show that 2ME2 plays a critical role in regulating RAS and RHR through downregulating AT1R and AGT. Additionally, the impact of 2ME2 on blood pressure is independent of sex in WKY rats. We also observed a substantial decrease in the heart weight of SHR treated with 2ME2, and bodyweight of 2ME2-treated groups. Collectively, our study suggests that 2ME2 has a cardioprotective effect, however, its safety and dosage in humans remain to be determined. Future studies are needed to confer its potential protection beyond CVD in postmenopausal women and age-matched men.

## Data Availability

The raw data supporting the conclusions of this article will be made available by the authors, without undue reservation.
